# Effects of “Bu Shen Huo Xue Decoction” on the Endometrial Morphology and Expression of Leukaemia Inhibitory Factor in the Rat Uterus during the Oestrous Cycle

**DOI:** 10.1155/2013/496036

**Published:** 2013-04-28

**Authors:** Xin Gong, Yanyan Yu, Qing Tong, Ying Ren, Zhe Jin

**Affiliations:** ^1^Reproductive Endocrinology Centre, Dongfang Hospital of Beijing University of Chinese Medicine, No. 6 Fangxingyuan 1 Qu, Fengtai District, Beijing 100078, China; ^2^Dongzhimen Hospital of Beijing University of Chinese Medicine, No. 5 Haiyuncang, Beijing 100700, China

## Abstract

The purpose of this study was to explore the positive effects of Bu Shen Huo Xue Decoction (BSHXF) on assisted reproduction. The study aimed to evaluate whether BSHXD could improve endometrial morphology and increase the expression of LIF in a gonadotrophin-releasing hormone agonists (GnRHa) long protocol-induced rat model during metestrus, diestrus, proestrus, and oestrus. The BSHXD group presented significantly increased endometrium thickness and decreased MVD compared with the GnRHa long protocol group. In addition, the expression of LIF was significantly higher in the BSHXD group. There were no significant differences between the control group and the BSHXD group in terms of MVD and LIF expression. These results suggested that BSHXD can improve the endometrium development, reduce the abnormal angiogenesis, and increase the expression of receptivity markers in a GnRHa long protocol-induced rat model during the oestrous cycle, which might result in an endometrial environment better suited for female reproduction.

## 1. Background

Infertility remains a prevalent disease worldwide, and its incidence is still increasing. Assisted reproductive technology (ART) is one current treatment. However, the fertility rate following ART is very low [1]. Impaired endometrial receptivity is a major limitation and reason for this low fertility.

It has been well documented that endometrial receptivity is a complex and multifactorial process [2] involving a good-quality embryo, a receptive endometrium, and the synchronisation between the developmental stages of the embryo itself [3]. Previous observations have demonstrated that when using a GnRHa long protocol, advanced endometrial maturation is present on the oocyte retrieval day of in vitro fertilisation (IVF), which may possibly result in a failure to correctly synchronise the timing between the embryo and a receptive endometrium [4]. Many studies have shown that the periovulatory uterine characteristics in ovarian stimulation treatment (OS) are considerably different compared with the natural cycle [4, 5]. It has been hypothesised that this change has already started by the follicular phase [5].

A large amount of evidence indicates that angiogenesis may play an important role during the implantation window [6–8], because the endometrial receptivity requires complex remodelling and angiogenesis to support foetal development [9–12]. Our previous small-sample study demonstrated that the GnRHa long protocol results in abnormal angiogenesis in the rat endometrium during the first oestrous cycle after OS. These results indicate that the side effects of the GnRHa long protocol treatment may trigger negative angiogenesis in the endometrium and impact later receptivity. The strong exposure of the endometrium to supraphysiological steroid hormone levels during the follicular phase might be responsible for this phenomenon [4].

In China, traditional Chinese medicine is widely used in assisted reproductive technology to enhance the success of IVF treatment. Bu Shen Huo Xue Decoction (BSHXD), a Chinese herbal formula, consists of *Placenta Hominis*, *Radix Rehmanniae Preparata*, *Radix Salviae Miltiorrhizae*, *Radix Angelicae Sinensis*, *Radix Dipsacus asperoides*, *Eucommia ulmoides*, *Dioscorea opposita*, *Flos Rosae Rugosae*, *Rhizoma Ligustici Chuanxiong*, and *Semen Coicis*. This formula is used to tonify the kidney, regulate the uterine function, and promote blood circulation according to traditional Chinese medicinal prescriptions. Numerous studies have reported that many traditional Chinese medicinal herbs are rich sources of compounds that regulate angiogenesis [13]. Our recent clinical observations suggest that BSHXD has a positive effect on angiogenesis in the endometrium. Therefore, the potential benefits of regulating angiogenesis and enhancing the success of IVF need to be thoroughly studied.

Microvessel density (MVD) is one of the most common methods of indirectly assessing angiogenesis factors [14]. The maximal expression of leukaemia inhibitory factor (LIF) is observed in the midsecretory phase of the menstrual cycle, coinciding with the time of implantation in both the human and murine endometria [15, 16]. The strong expression of LIF is more likely to initiate a pregnancy than weak LIF during the luteal phase prior to IVF treatment in humans [17, 18]. LIF is one of very few cytokines to be a critical factor for implantation [19]. LIF null mutation female mice are infertile because of the failure of implantation [20] and LIF is significantly reduced in the endometria of infertile women [21].

The primary aim of this study was to explore whether BSHXD ameliorates the side effects of the GnRHa long protocol on endometrium histology, endometrium receptivity cytokines, and endometrial angiogenesis in a GnRHa long protocol-induced rat model. In addition, this study aimed to provide evidence of the usefulness of BSHXD in assisted reproduction.

## 2. Methods

### 2.1. Animals

SD (Sprague-Dawley) rats of 9-10 weeks of age were used for the experiments. All the procedures were performed according to the guidelines of the Beijing University of Chinese Medicine Animal Care and Use Committee. The rats were kept under standard 12 h light and 12 h dark conditions and under controlled temperature (23 ± 3°C) with 45%–65% humidity.

### 2.2. Treatment

Oestrus was identified by vaginal smear. Only the rats with regular cycles were used in the study. Suitable rats were randomly allocated into three groups: control, GnRHa long protocol, and BSHXD.

The animals in the GnRHa long protocol group were given 1 mL/100 g of distilled water for 12 days and then treated using the GnRHa long protocol. Briefly, a GnRH agonist (1.5 *μ*g/100 g bw/day) (triptorelin, Diphereline, France) was i.p. injected from the 3th to 9th days of oestrous. The pregnant mare's serum gonadotropin (40 IU/100 g bw) (PMSG, China) was i.p. injected on the 9th day of oestrous, followed by the injection of hCG (40 IU/100 g) (Human Chorionic Gonadotropin, China) 28 h later.

BSHXD granules were provided by the Pharmacy Department of Dongfang Hospital of Beijing University of Chinese Medicine. The BSHXD granules contain equal weights of the ingredients of the BSHXD formula: *Placenta Hominis* 10 gram, *Radix Rehmanniae Preparata* 15 gram, *Radix Salviae Miltiorrhizae* 10 gram, *Radix Angelicae Sinensis* 12 gram, *Radix Dipsacus Asperoides* 15 gram, *Eucommia ulmoides* 12 gram, *Dioscorea opposita* 15 gram, *Flos Rosae Rugosae* 6 gram, *Rhizoma Ligustici Chuanxiong* 6 gram, and *Semen Coicis* 12 gram. The granules were dissolved in 200 mL of distilled water and kept at 2–8°C until use. The animals in the BSHXD group were given the drugs 1 mL/100 g daily for 12 days and then were subjected to the GnRHa long protocol treatment as the GnRHa long protocol group.

The rats in the control group were given distilled water for 12 days, followed by injections with saline at the same time and volume as those used in the GnRHa long protocol group.

### 2.3. Tissue Collection and Preparation

The whole uteri were collected from the GnRHa long protocol group and the BSHXD group on days 2, 3, 4, and 5 after hCG injection, and the uteri of the control group were collected on days 2, 3, 4, and 5 after ovulation. Day 2 was the day of metestrus. Day 3 was the day of diestrus. Day 4 was the day of proestrus. Day 5 was the day of oestrus. The tissue was divided into 2 parts after being rinsed with cold saline. One part was fixed in 4% paraformaldehyde and then embedded in paraffin for HE and immunohistochemical assays. The other part was stored at −80°C for later Western blot analysis.

### 2.4. Haematoxylin Eosin (HE) Staining

The paraffin-fixed tissues were divided into the 4 *μ*m. After dewaxing, rehydration, and staining in Harris haematoxylin, the slides were counterstained in eosin-phloxine. The morphological changes were captured with a digital camera (Olympus, Inc., Tokyo, Japan).

### 2.5. Immunohistochemistry for CD34 and LIF

The paraffin-fixed tissues were divided into 4 *μ*m sections. After dewaxing, rehydration, and blocking, the slides were incubated with the following primary antibodies: CD34 antibody (AF-4117, R&D Systems, USA) at a dilution of 1 : 39 and LIF antibody (sc-1336, Santa Cruz Biotechnology, USA) at a 1 : 200 dilution overnight at 4°C. After washing with PBS, the tissues were incubated with secondary antibodies for 25 minutes, followed by incubation with a DBA Kit (ZLI-9018, ZSGB-BIO, China). The negative controls were treated with the same procedure except with the PBS during the primary antibody incubation step.

The staining intensity of each slide was graded (0, absence; 1, weak; 2, moderate; 3, strong) by two examiners in a blinded fashion and assessed with the HSCORE. The HSCORE was calculated as follows:

(1) $$\begin{array}{c} \text{HSCORE}=P_{i}\left(\;i+1\right), \end{array}$$

where *i* is the staining intensity and *P*_*i*_ is the percentage of stained glandular epithelium cells at each level of intensity.

### 2.6. Density of Microvessels

Microvessels densities were viewed at 400x magnification (40x objective lens and 10x ocular lens; 0.24 mm^2^/field). The images were captured with a digital camera (Olympus, Inc., Tokyo, Japan). For each section, at least 5 random fields were selected to determine the average vessel density within the uterus.

The number of CD34-positive vessels was quantified using Diagnostic Instruments Spot-II digital software (Diagnostic instruments, Inc., USA). The microvessel density was calculated as the number of CD34-positive vessels/(40 × 0.24 mm^2^).

### 2.7. Western Blot for LIF

Rat endometrium tissue was homogenised and lysed in RIPA Lysis Buffer (C1053, Applygen, China) and proteinase inhibitor (P1265, Applygen, China). The protein concentration was quantified with bicinchoninic acid (BCA) (P1511, Applygen, China). The protein was used for Western blot with an LIF primary antibody (sc-1336, Santa Cruz Biotechnology, Europe) at a 1 : 500 dilution and incubated overnight at 4°C. After incubation, the membranes were washed three times with TBS-T and then incubated with the secondary antibody at a dilution of 1 : 2500 at room temperature for 1 h. The blots were visualised with Super ECL Plus Detection Reagent (P1010, Applygen, China). The ECL signals were detected with Quantity One software (Bio-Rad). GAPDH (ab8245, Abcam, UK) was used as an internal control to validate the amount of protein loaded onto the gels.

### 2.8. Statistical Analysis

The data are shown as the mean ± SEM. The Mann-Whitney *U* test was used to compare the two groups. Significance was set at *P* value < 0.05. Graphs of the data were produced using Excel software.

## 3. Results

### 3.1. Endometrial Thickness

**Figure 1:**
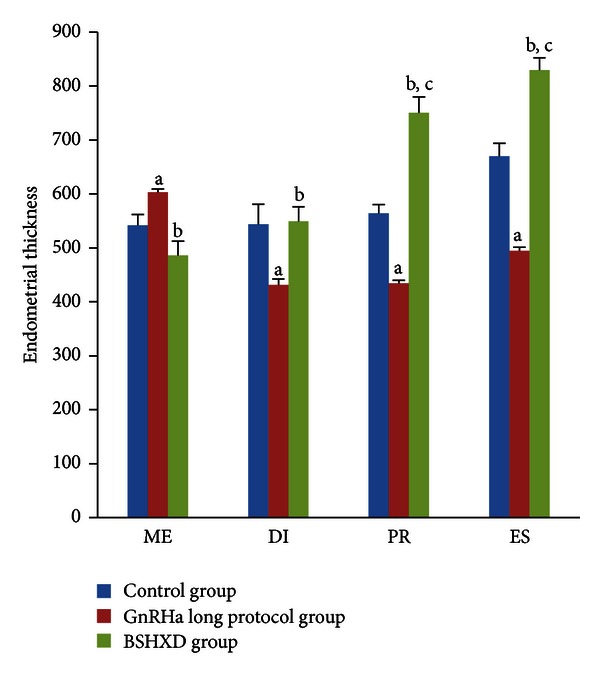
HE staining to evaluate the rat endometrial thickness. Comparison of thickness of rat uteri during metestrus (ME), diestrus (DI), proestrus (PR), and estrus (ES). (a) *P* values < 0.01 compared with the control group; (b) *P* values < 0.01 compared with the GnRHa long protocol group; (c) *P* values < 0.01 compared with the control group. The bar graphs represent daily data from 6 different animals.

The outcome of endometrial thickness was obtained by two independent observers blinded to treatment. The morphological change parameters and data are shown in Figure 1 and Table 1. The endometrial thickness of the GnRHa long protocol group was thicker than that of the control group and BSHXD group in metestrus (*P* values of 0.004), but the GnRHa long protocol group endometrial thickness was thinner than the control group mean (*P* values of 0.009, 0.002, and 0.002, resp.) and the BSHXD group mean (*P* values of 0.002, 0.002, and 0.002, resp.) in diestrus, proestrus, and oestrus periods. There was no significant difference between the BSHXD group and the control group in metestrus and diestrus (*P* values of 0.24 and 0.82, resp.). The thickness in the BSHXD group was significantly thicker than that in the control group in proestrus and oestrus (*P* values of 0.002 and 0.002, resp.).

### 3.2. Expression of Microvessel Density (MVD)

**Figure 2:**
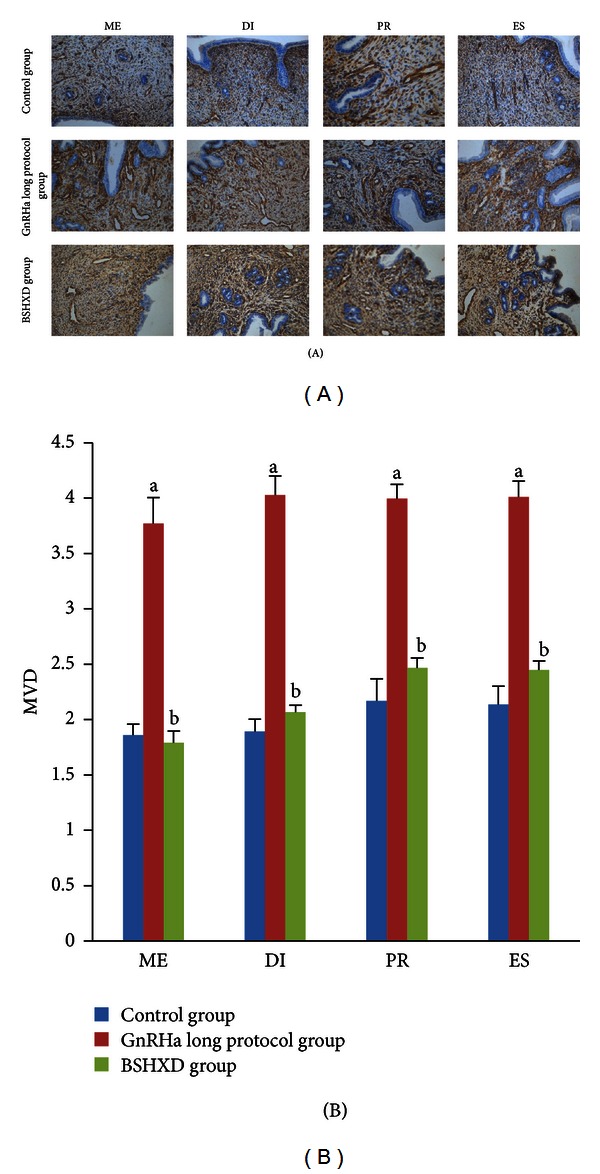
Expression of microvessel density (MVD) during metestrus (ME), diestrus (DI), proestrus (PR), and oestrus (ES). (A) Comparison of the MVD of rat uteri during metestrus (ME), diestrus (DI), proestrus (PR), and oestrus (ES). Magnification: 200x. (B) The number of CD34-positive vessels was quantified. The bar graphs represent daily data from 6 different animals. (a) *P* values < 0.01 compared with the control group; (b) *P* values < 0.01 compared with the GnRHa long protocol group.

Accompanying the GnRHa long protocol treatment-induced downregulation of LIF expression, there was a significant increase in endometrial MVD in rats (MVD: 3.77 ± 0.24 in metestrus, 4.03 ± 0.17 in diestrus, 3.99 ± 0.13 in proestrus, and 4.01 ± 0.14 in oestrus) (Figure 2). Related to the GnRHa long protocol group, no increase was observed in the control and the BSHXD groups in metestrus, diestrus, proestrus, and oestrus (*P* values of 0.002, 0.002, 0.002 and 0.002, resp.).

### 3.3. Expression of Endometrial LIF as Evaluated by Immunohistochemistry

**Figure 3:**
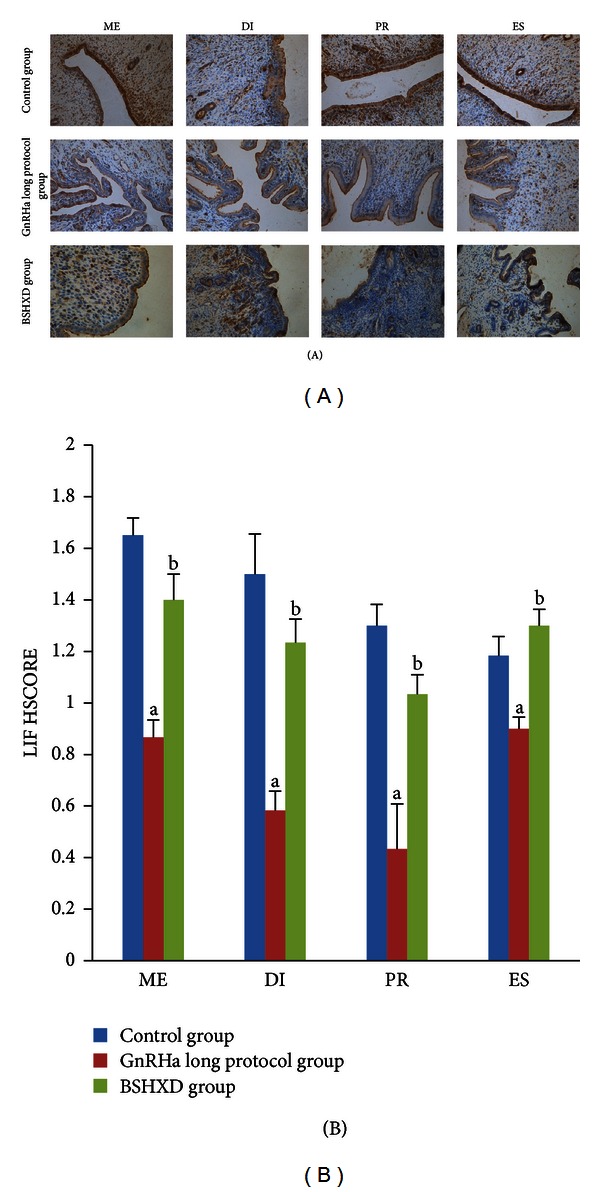
LIF immunohistochemical staining in the endometrium. (A) Immunohistochemical staining to evaluate the expression of endometrial LIF during metestrus (ME), diestrus (DI), proestrus (PR), and oestrus (ES). Magnification: 200x. (B) HSCORE for the immunohistochemical staining intensity of the endometrial LIF during metestrus (ME), diestrus (DI), proestrus (PR), and oestrus (ES). The bar graphs represent daily data from 6 different animals. (a) *P* values < 0.05 compared with the control group; (b) *P* values < 0.05 compared with the GnRHa long protocol group.

LIF immunostaining was predominantly detected in glandular and luminal epithelial cells in rat endometrium. The LIF staining intensity in the GnRHa long protocol group was weaker than that in the control group in metestrus, diestrus, proestrus, and oestrus (*P* values of 0.002, 0.002, 0.002, and 0.015, resp.) and weaker than that of the BSHXD group (*P* values of 0.002, 0.002, 0.015, and 0.002, resp.). During the oestrus cycle, there were no significant differences between the control group and BSHXD group in metestrus, diestrus, proestrus, and oestrus (*P* values of 0.093, 0.24, 0.065, and 0.31, resp.). The staining intensities are depicted in Figure 3.

### 3.4. Western Blot Expression of Endometrial LIF

**Figure 4:**
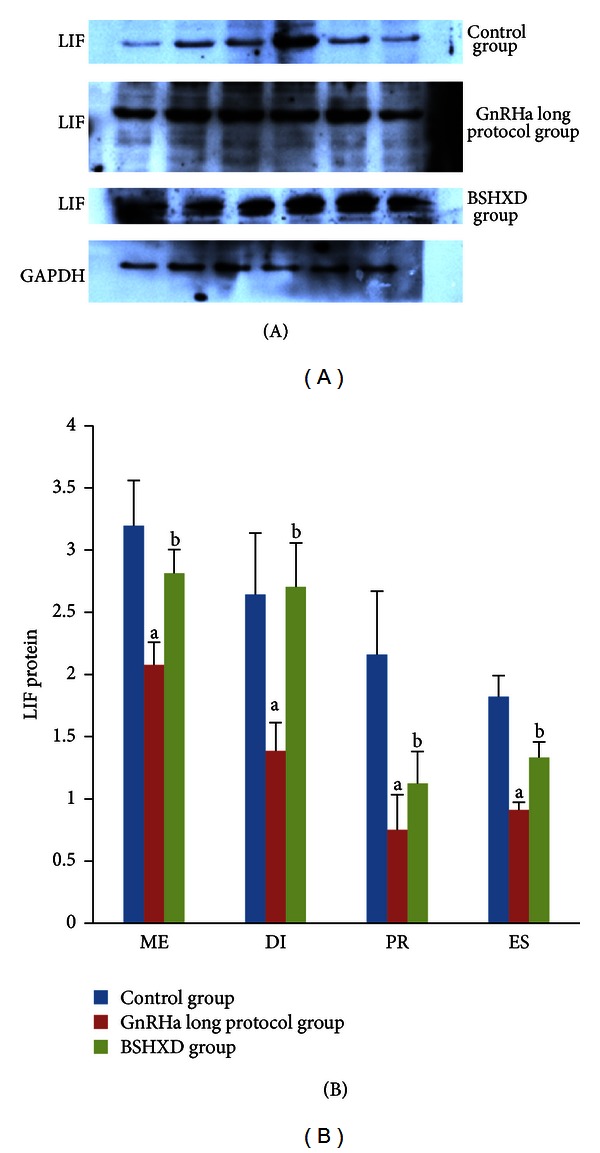
Expression of endometrial leukaemia-inhibitory factor (LIF) protein during metestrus (ME), diestrus (DI), proestrus (PR), and oestrus (ES). The bar graphs represent daily data from 6 different animals. (a) *P* values < 0.05 compared with the control group; (b) *P* values < 0.05 compared with the GnRHa long protocol group.

Consistent with the results of the immunohistochemical staining, the LIF protein expression trends were confirmed by Western blot analysis (Figure 4). GAPDH was used as an internal loading control in each lane. Normalised with the GAPDH expression level, the expression of LIF protein in the GnRHa long protocol group was lower than that in the control group (*P* values of 0.026, 0.004, 0.015, and 0.009, resp.) and the BSHXD group (*P*  values of 0.041, 0.015, 0.041, and 0.015, resp.) in metestrus, diestrus, proestrus, and oestrus. There were no significant differences between the control group and BSHXD group (*P* values of 0.485, 0.699, 0.065, and 0.065, resp.).

## 4. Discussion

The present study is a pilot, prospective, randomized, and controlled comparison study of the effects of BSHXD on the endometrial morphology and expression of LIF and MVD in a GnRHa long protocol-induced rat model during the oestrus cycle. The process of implantation only takes place during a limited “implantation window.” This window is a restricted period of endometrial receptivity between days 4 and 6 of pregnancy in rats [22] and between days 20 and 24 of a regular menstrual cycle (day LH+7 to LH+11) in humans [23]. Implantation involves a complex sequence of signalling events that are crucial to the pregnancy. Most of these identified molecular mediators are under the influence of ovarian hormones [24, 25]. However, the GnRHa long protocol will result in the endometrium exposure to supraphysiological steroid hormone levels. The dosage in our experiment produced thin and impaired endometria in our rat models and abnormal expression of LIF and MVD, which may impact later endometrial implantation capacity. Predictably, the GnRHa long protocol impaired the endometrium and implantation at a very early stage even at the follicular phase. Our observations are in agreement with a previous study that confirmed the negative impact of GnRHa ovarian stimulation treatment [26–28], which may cause a relatively low rate of implantation despite advances in ATR [29]. Indeed, the average implantation rate in IVF is approximately 25% [30].

Endometrial thickness is an important bioassay. According to our present study results, the endometrial thickness in the GnRHa long protocol group was significantly lower than that of the control and BSHXD groups during diestrus, proestrus, and oestrus. These results suggest that the uterus is quite sensitive to hormonal change. Although some researchers have argued that histological endometrial data do not predict and influence the reproductive failure [31], increased endometrial thickness is associated with improved pregnancy rates [32]. Some studies have revealed that below a certain thickness cut-off limit, pregnancy will not be achieved [33–35]. We observed that BSHXD had positive effects on endometrial morphology in rats after they were subjected to a GnRHa long protocol treatment. There were no significant differences between the control group and BSHXD group in metestrus and diestrus. Compared with the control group, the BSHXD group presented enhanced endometrial thickness during the proestrus and oestrus stages. Thus, we suggest that BSHXD may have the capacity to stimulate the growth of the endometrium.

Because adequate blood flow to the embryo is critical for normal growth, it is not surprising that angiogenesis plays an important role during implantation. Dysregulated endometrial angiogenesis underlies infertility [36–38]. Therefore, appropriate angiogenesis is central to implantation and pregnancy. There was an approximately 2-fold increase in MVD in the GnRHa long protocol group compared with the control and BSHXD groups. However, there was no difference between the control group and BSHXD group. These results demonstrate that pretreatment with BSHXD can significantly ameliorate the negative effects of the GnRHa long protocol on MVD. We suggest that BSHXD may regulate angiogenesis during the oestrous cycle.

The present study demonstrated that BSHXD improved the expression of LIF protein in rat uteri during the oestrous cycle. LIF is a biomarker that is largely accepted as an indicator of endometrial receptivity both in humans, mice, and rats. LIF-deficient mice are mostly infertile [20], clearly indicating the important role. Our research confirmed the result of a previous study that indicated that LIF protein is maximally expressed in the murine endometrial glandular epithelium [39, 40]. Our results demonstrated that the GnRHa long protocol affected cytokine production at a very early stage, even before the “implantation window,” because the staining intensity of LIF in the GnRHa long protocol group was weaker than the other two groups (*P* values < 0.05). However, there were no significant differences between the control and BSHXD groups (*P* values > 0.05). Our results indicate that BSHXD improves the LIF expression and brings its level closer to normal, which may help improve the later endometrial implantation capacity.

Most TCM remedies are formulated using individual herbs in combination because different herbs are thought to increase therapeutic efficacy and reduce adverse effects simultaneously through multiple targets and biological pathway [41]. The biological mechanisms underlying the effect of BSHXD on assisted reproduction remain unclear. However, our study demonstrated that the positive effects of BSHXD may be associated with the following: (a) increasing endometrial thickness, (b) regulation of angiogenesis to improve the endometrial environment, and (c) modulating cytokines that are associated with the pregnancy rates of IVF. According to the principles of TCM, BSHXD can nourish the uterus and adjust its function.

In conclusion, BSHXD improved the uterine environment by advancing endometrial development, reducing abnormal angiogenesis, and increasing expression of the protein receptivity marker LIF during the oestrous cycle. BSHXD may be useful aid for female reproduction. A further clinical evaluation needs to be conducted to confirm these results in human subjects.

## Figures and Tables

**Table 1:** Comparison of the endometrial thickness during the oestrous cycle.

Endometrial thickness (*μ*m)	Control group	GnRHa long protocol group	BSHXD group	*P* valve		
D2 (Metestrus)	541.97 ± 19.43	602.92 ± 6.39	485.98 ± 26.53	0.00^a^	0.00^b ^	0.24^c^
D3 (Diestrus)	543.61 ± 37.37	431.48 ± 10.82	549.07 ± 27.09	0.00^a^	0.00^b^	0.82^c^
D4 (Proestrus)	563.86 ± 16.68	434.05 ± 5.77	750.08 ± 29.23	0.00^a^	0.00^b^	0.00^c^
D5 (Oestrus)	669.76 ± 24.10	494.25 ± 6.52	829.40 ± 22.65	0.00^a^	0.00^b^	0.00^c^

^a^Control group versus the GnRHa long protocol group.

^b^BSHXD group versus the GnRHa long protocol group.

^c^Control group versus the BSHXD group.
